# Sex differentials in relationships between functional fitness and cognitive performance in older adults: a canonical correlation analysis

**DOI:** 10.1038/s41598-018-22475-7

**Published:** 2018-03-07

**Authors:** Yan Guo, Mei Yang, Yaqiong Yan, Liang Wang, Jie Gong

**Affiliations:** 1 Wuhan Centers for Disease Prevention and Control, Wuhan, China; 2 Department of Maternal and Child Health, School of Public Health, Wuhan University of Science and Technology, Wuhan, China; 3 Hubei Province Key Laboratory of Occupational Hazard Identification and Control, Wuhan University of Science and Technology, Wuhan, China

## Abstract

This study aimed to explore the sex differentials in correlations between functional fitness (FF) and cognitive impairment (CI) in older adults without dementia. A community-based cross-sectional study was conducted using 2096 adults more than 65 years of age. The Senior Fitness test and Mini-mental State Examination (MMSE) were used to measure FF and cognitive performance. Canonical correlation analysis (CCA) was performed to evaluate the relationships between FF and MMSE. Our results confirmed the relationships between FF and CI, furthermore FF and MMSE were significantly different between men and women (*P* < 0.05). CCA results showed overall FF was positively correlated with overall MMSE in both men (canonical coefficient = 0.37, *P* < 0.0001) and women (first canonical coefficient = 0.42, *P* < 0.0001; second canonical coefficient = 0.17, *P* = 0.004). Among men, 30s-arm curl and language were most highly correlated with FF and MMSE, respectively. Whereas among women, 30s-arm curl and eight-foot up-and-go were most highly correlated with FF, and orientation and recall were most highly correlated with MMSE. In conclusion, there was a sex difference in the relationships between FF and MMSE, which facilitated generating insight into cognitive performance improvement from the perspective of FF enhancement by sex. Prospective studies are needed to explore the causality between FF and cognitive performance.

## Introduction

With the progress of civilization and industrialization, the aging population dramatically increases worldwide. In China, there were 131.61 million older adults (≥65 years old) as of 2015. Numerous problems manifest throughout the aging process. Dementia and cognitive impairment (CI) have become one of the burdensome public health issues nowadays^1^. As projected by Alzheimer’s Disease International in 2015, the number of dementia patients worldwide was 46.8 million in 2015 and is expected to double every 20 years and arrive at 131.5 million by 2050^2^.

Earlier detection and intervention have been suggested as the most cost-effective measures to prevent or slowing the onset of dementia^3^. Hence, it is necessary to identify potentially modifiable lifestyle-related markers in the earliest phase of the disease. CI is characterized by declines in memory, attention and cognitive function, which greatly affects the quality of life. Mini-mental state examination (MMSE) is a widely used method to evaluate the cognitive state. Functional fitness (FF) is defined as having the physical capacity to perform normal daily activities safely and independently without undue fatigue. A review of the existing literature indicated that decreased FF was a risk factor of dementia, and a high level of FF might reduce the risk of this disease^4–6^. Furthermore, few studies have studied the associations between FF and cognitive status in older adults free from dementia. A community-based study conducted in the Japanese population found that each FF component had potential as a single marker of low cognitive function^7^. Meanwhile, a study conducted in the Korean population showed that only eight-foot up-and-go and 6-min walk test domains were significantly associated with global cognitive performance^8^. In addition, two other recent studies indicated that gait speed was related to cognitive function in the pre-dementia stage^9,10^. However, it is not known whether FF is associated with cognitive function in Chinese older adults without dementia.

Furthermore, Chinese oldest old (i.e., people aged 80 and older) women are at a higher risk for CI than men due to sex disparities^11^. Both MMSE and FF are multi-dimensional concepts. Thus it is necessary for health education to examine the sex differentials in relationships between FF and cognitive function, and explore the key sub-dimensions that influence their overall relationships. To date, few studies have performed such examinations.

Our study aimed to explore the relationships between FF and CI, between sex, and apply canonical correlation analysis (CCA) to identify the key sub-dimensions of FF and cognitive function influencing their overall relationships in Chinese older adults free from dementia.

## Results

The average age (72.79 ± 5.10 vs. 72.66 ± 5.28; *t* = 0.60, *P* = 0.55) and body mass index (BMI, 24.28 ± 3.42 vs. 24.66 ± 3.79; *t* = 1.88, *P* = 0.06) for men and women were not significantly different. Compared with female older adults, males had a higher rate of smoking (34.34% vs. 2.16%, *P* < 0.0001).

### Associations between FF and CI by sex

Male older adults with CI performed more poorly on all FF tests when compared with non CI older male adults, having significantly fewer 30s-chair stands, 30s-arm curls and 2 min-steps, and longer time to complete 8-foot up-and-go, shorter chair sit-and-reach and back scratch distance(all *P* < 0.05). Similarly, female older adults with CI also performed more poorly on all FF tests when compared with non-CI older female adults. The detailed values are presented in Table 1.

**Table 1:** Functional fitness of the study population with and without cognitive impairment by sex.

	Men (n = 1031)			Women (n = 1065)		
	CI (n = 217)	Non-CI (n = 814)	*P*	CI (n = 457)	Non-CI (n = 608)	*P*
30 s Chair stand	11.89 ± 5.78	14.82 ± 6.33	<0.0001	11.59 ± 5.31	13.88 ± 5.53	<0.0001
Chair sit-and reach	−6.86 ± 10.33	−4.05 ± 8.65	0.0004	−5.31 ± 9.71	−1.52 ± 7.90	<0.0001
30s-arm curl	13.82 ± 7.10	18.66 ± 9.59	<0.0001	13.59 ± 6.86	18.50 ± 9.13	<0.0001
2-min step	67.20 ± 40.76	83.88 ± 38.52	<0.0001	62.54 ± 35.19	80.26 ± 36.10	<0.0001
Back scratch	−17.49 ± 12.68	−12.53 ± 11.71	<0.0001	−14.72 ± 12.03	−10.85 ± 12.87	<0.0001
8 foot up-and-go	10.19 ± 4.75	8.55 ± 3.93	<0.0001	10.45 ± 10.04	8.69 ± 8.34	<0.0001

*P* was calculated using unpaired student’s *t*-test.

### Sex differentials of functional fitness and mini-mental state examination

As summarized in Table 2, male older adults had better performance on all FF tests when compared with females, except one. These included significantly higher 30s-chair stands, 30s-arm curls and 2 min-steps, and less time to complete eight-foot up-and-go, but shorter chair sit-and-reach distance (all *P* < 0.05). Furthermore, total MMSE score was significantly higher in male older adults than females (26.02 ± 4.44 vs. 23.40 ± 5.85; *t* = 11.51, *P* < 0.0001). In addition, for each domain of MMSE including orientation, memory, attention, recall, language, male older adult scores were significantly higher than females (all *P* < 0.05).

**Table 2:** Clinical Characteristics of the study population by sex.

	Total (n = 2096)	Men (n = 1031)	Women (n = 1065)	*P*
**Senior functional fitness test**				
30 s Chair stand (n)	13.55 ± 5.98	14.22 ± 6.33	12.91 ± 5.56	<0.0001
Chair sit-and-reach (cm)	−3.87 ± 9.03	−4.43 ± 9.09	−3.14 ± 8.91	0.0002
30s-arm curl (n)	17.08 ± 9.00	17.69 ± 9.34	16.47 ± 8.61	0.002
2 min step (n)	76.62 ± 38.33	80.49 ± 39.54	72.85 ± 36.76	<0.0001
Back scratch (cm)	−13.07 ± 12.38	−13.60 ± 12.09	−12.56 ± 12.65	0.06
Eight-foot up-and-go (s)	9.17 ± 4.34	8.89 ± 4.16	9.44 ± 4.48	0.004
**Mine-mental State examination**				
Total score	24.69 ± 5.36	26.02 ± 4.44	23.40 ± 5.85	<0.0001
Orientation	8.68 ± 2.08	9.10 ± 1.65	8.28 ± 2.37	<0.0001
Memory	2.77 ± 0.62	2.82 ± 0.57	2.73 ± 0.66	0.001
Attention	3.51 ± 1.79	3.97 ± 1.52	3.06 ± 1.92	<0.0001
Recall	2.09 ± 1.05	2.21 ± 0.98	1.97 ± 1.09	<0.0001
Language	7.67 ± 1.38	7.94 ± 1.30	7.41 ± 1.41	<0.0001

*P* was calculated using unpaired student’s t-test.

### Canonical correlation analysis

FF was assigned x variables (x_1_ = 30s-chair stand, x_2_ = chair sit-and-reach, x_3_ = 30s-arm curl, x_4_ = 2 min-step, x_5_ = back scratch, x_6_ = eight-foot up-and-go), and MMSE was assigned y variables (y_1_ = orientation, y_2_ = memory, y_3_ = attention, y_4_ = recall, y_5_ = language). There were low to moderated correlations within and between the 6 FF variables and 5 MMSE variables in men and women respectively (Table 3).

**Table 3:** Correlations between the sub-dimensions of functional fitness and mini-mental state examination for men and women.

Variables	y_1_	y_2_	y_3_	y_4_	y_5_
**Men**					
x_1_	0.12	0.00	0.17	0.11	0.20
x_2_	0.06	0.00	0.13	0.08	0.16
x_3_	0.16	0.00	0.24	0.09	0.24
x_4_	0.12	−0.04	0.16	0.09	0.18
x_5_	0.15	0.03	0.17	0.11	0.18
x_6_	−0.10	0.04	−0.16	−0.08	−0.10
**Women**					
x_1_	0.21	0.07	0.15	0.13	0.15
x_2_	0.21	0.06	0.18	0.13	0.19
x_3_	0.33	0.13	0.27	0.15	0.25
x_4_	0.23	0.05	0.23	0.22	0.21
x_5_	0.17	0.07	0.17	0.16	0.16
x_6_	−0.18	−0.01	−0.13	−0.17	−0.09

*r* was calculated by Pearson correlation test.

x_1_ = 30 s chair stand, x_2_ = chair sit-and-reach, x_3_ = 30s-arm curl, x_4_ = 2 min step, x_5_ = back scratch, x_6_ = eight-foot up-and-go; y_1_ = orientation, y_2_ = memory, y_3_ = attention, y_4_ = recall, y_5_ = language.

The results of CCA yielded five canonical functions. For men, only the first canonical function (canonical coefficient = 0.37, *P* < 0.0001) was statistically significant. But for women, both the first (canonical coefficient = 0.42, *P* < 0.0001) and second (canonical coefficient = 0.17, *P* = 0.004) canonical functions were significant. According to the standardized canonical coefficients between variables and the canonical functions (V in FF and W in MMSE), the following equations were established.

V_1_ (men) = 0.198x_1_ + 0.167x_2_ + 0.528x_3_ + 0.228x_4_ + 0.365x_5_ − 0.047x_6_

W_1_ (men) = 0.151y_1_−0.393 y_2_ + 0.470y_3_ + 0.157y_4_ + 0.583y_5_

V_1_ (women) = 0.008x_1_ + 0.241x_2_ + 0.588x_3_ + 0.334x_4_ + 0.328x_5_ − 0.021x_6_

W_1_ (women) = 0.545y_1_−0.044y_2_ + 0.268y_3_ + 0.206y_4_ + 0.244y_5_

V_2_ (women) = −0.075x_1_ + 0.173x_2_ + 0.737x_3_ − 0.273x_4_ − 0.193x_5_ + 0.817x_6_

W_2_ (women) = 0.045y_1_ + 0.581 y_2_ − 0.054y_3_ − 1.028y_4_ + 0.585y_5_

Furthermore, V_1_ and W_1_ could explain 38.04% and 44.08% of FF and MMSE variance in men respectively. As for women, V_1_ and V_2_ could explain 34.86% and 13.79% FF variance, whereas W_1_ and W_2_ could explain 49.84% and 12.15% MMSE variance respectively.

**Figure 1:**
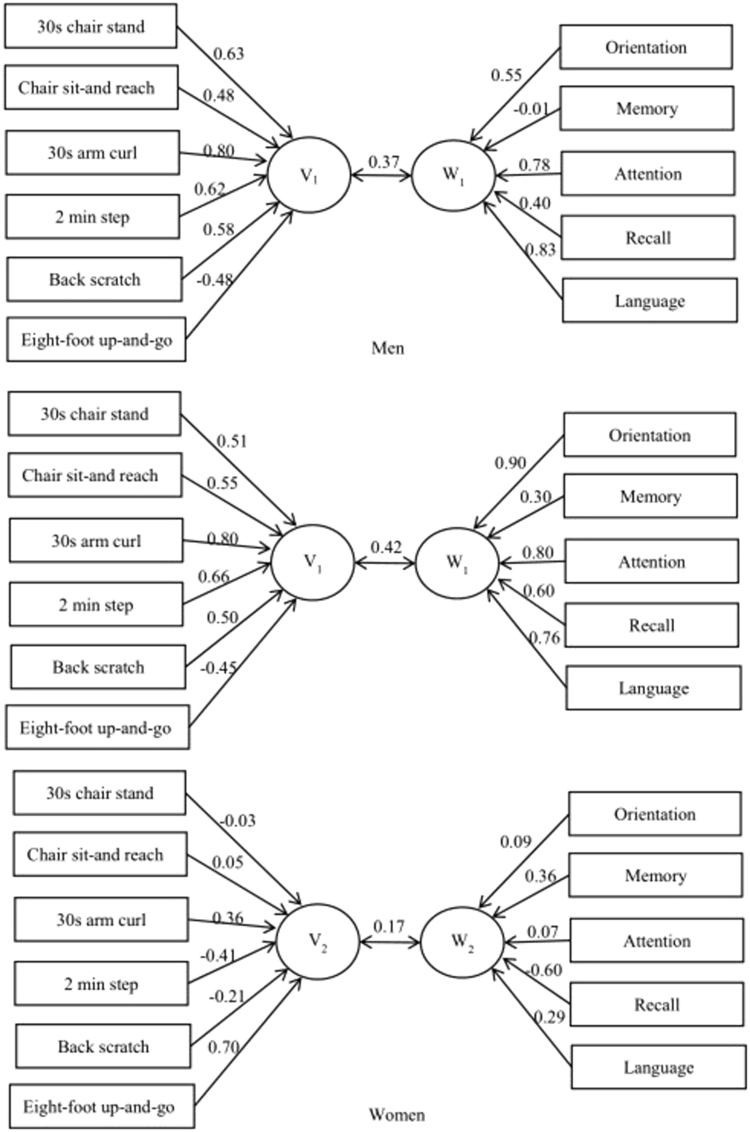
Structure coefficient of canonical factors. For male older adults, 30s-arm curl were most highly correlated with V1 in FF, and Language were most highly correlated with W1 in MMSE. Among the female older adults, 30s-arm curl (rs = 0.80) and orientation (rs = 0.90) were most highly correlated with V1 and W1; eight-foot up-and-go (rs = 0.70) and recall (rs = −0.60) were most highly correlated with V2 and W2, respectively.

As shown in Fig. 1, for male older adults, the structure coefficients showed that except for eight-foot up-and-go, other variables were positively associated with FF, indicating that higher frequency of chair stand, step, arm curl, as well as shorter chair sit-and-reach distance, back scratch distance and less time to complete eight-foot up-and-go were associated with higher FF performance. Moreover, the 30s-arm curl (*r*_*s*_ = 0.80) were most highly correlated with V_1_ in FF, and Language (*r*_*s*_ = 0.83) were most highly correlated with W1 in MMSE. Among the female older adults, the 30s-arm curl (*r*_*s*_ = 0.80) and orientation (*r*_*s*_ = 0.90) were most highly correlated with V_1_ and W_1_ respectively. In addition, the eight-foot up-and-go (*r*_*s*_ = 0.70) and recall (*r*_*s*_ = −0.60) were most highly correlated with V_2_ and W_2_.

## Discussion

Exploring modifiable lifestyle-related markers of pre-dementia cognitive function was expected to be of value to promote earlier detection and intervention measures in community-based settings^12^. The results of our study confirmed the relationships between FF and CI both in male and female older adults free from dementia. In addition, positive moderate correlations between overall FF and overall MMSE, evaluated by CCA, were calculated. Muscle strength and flexibility, agility/dynamic balance, aerobic endurance all demonstrated significant contributions to the relationships between overall FF and overall MMSE. Namely, older adults with higher FF generally had better cognitive conditions, which is consistent with previous studies^8,13–15^. Furthermore, Narazaki *et al*. found handgrip strength, leg strength, sit-to-stand rate, gait speed and one-leg stand time had significant associations with global cognitive function in Japanese community-dwelling older adults without apparent cognitive problems^7^. Similar results have also been demonstrated in cognitive impairment populations. For example, Lee SH *et al*. conducted a cross-sectional study which enrolled 500 Korean older adults and found those who had mild cognitive impairment had significantly lower levels of physical fitness, especially upper body flexibility and agility/dynamic balance^15^. Moreover, the results from EPIDOS cohort study showed that there was a negative association of five-times-sit-to-stand with global cognitive performance, which meant the older adults with CI had poor agility and functional mobility^16^.

In the present study, the CCA results also showed that FF was associated more with orientation, attention and language, but less with memory, especially for men, which was opposite to our expectation. A recent study showed that in young adults, better aerobic fitness was associated with hippocampal viscoelasticity that mediated the benefits of fitness on memory function^17^. In addition, another study conducted in older adults at risk for Alzheimer’s disease showed that for men, significant positive associations were observed between cardiorespiratory fitness and memory, but not for women^18^. These inconsistencies might arise from the different age, mental/cognitive state of the populations studied, different evaluation methods of fitness and memory and so on.

It was well known that men owned better physical strength and women owned better body tenacity^19^, which were consistent with our FF results. Moreover, we also found that men performed better than women in each domain of MMSE. A growing body of research presented that Chinese women were significantly disadvantaged in cognitive functioning in old age^11,20–22^, as well as in the oldest old^11^.Furthermore, similar with our results (the prevalence of CI: men vs. women = 21.05% vs. 42.91%), Yu and colleagues also reported a large sex disparities in the prevalence of both sever and mild CI among those aged more than 65 years old in Shanghai, China^20^. Expect for the difference of physical structure, sex was one factor that hinted at the differences in gender roles, opportunities and obligations which conditioned the experiences of men and women throughout their life course. Lack of mental stimulation through high-skilled occupation and a large social network, which were associated with cognitive development and maintenance^23^, might contribute to the lower MMSE in older women.

In addition, it was worth noting that the key sub-dimensions of FF and MMSE were obviously different for male and female. For men, 30s-arm curl were most highly correlated with FF, whereas, among the female, 30s-arm curl and eight-foot up-and-go were most highly correlated with FF. Furthermore, the characteristics of FF were quite different between men and women. So for male and female, there should be different intervention programs for the improvement of FF. In addition to upper body strength, women should focus on the promotion of aerobic endurance and balance. Furthermore, for men language were most highly correlated with MMSE, but for women orientation and recall were most highly correlated with MMSE. That means, different sex may have different manifestations of CI. So in the early stage of cognitive degradation, focusing on different functional domains depending on sex might reduce the workload of primary health care workers.

Senior Fitness Test (SFT) required refined brain control for initiation of the tasks, recruitment of muscles and motor coordination. Hence, the concurrent deterioration of the brain regions responsible for cognitive and physical performance might be one possible mechanism explaining the observed association^24–26^. Moreover, other researchers found that changes in the brain activation of older adults with aerobic exercise intervention have been accompanied by improvement in cognitive function^27,28^. Taking into account the results of our study, SFT was simple and required no clinical resources or devices, so physical fitness measures was of practical value for identifying and monitoring pre-clinical cognitive impairment in community-based regular checkups.

Even with a larger sample size, our study had some limitations. Firstly, this study was a cross-sectional study which could not clarify the causality between FF and MMSE. Secondly, we did not evaluated potential confounders of FF and MMSE, such as the years of education, which might limit the interpretation of the results. Therefore, larger and prospective studies are needed to further explore the association between FF and cognitive status in Chinese older adults free from dementia.

In summary, our results confirmed the relationships between FF and CI in Chinese older people free from dementia. Furthermore, the present study first demonstrated the correlations between six FF and five MMSE sub-dimensions using CCA and revealed positive and moderate relationships between FF and MMSE. The main FF components that influence the relationships between FF and MMSE in men are upper and lower body strength, whereas in women, the components are upper body strength, aerobic endurance and agility/dynamic balance. Hence, it is necessary to promote sex-specific fitness interventions to improve cognitive status among Chinese older adults.

## Methods

### Study population

Our study was designed as a cross-sectional study based on community. Participants were selected by multi-stage stratified random sampling in Wuhan city, Hubei province, central China, during December 2015 to May 2016. Firstly, 7 districts were randomly selected from the 17 districts of Wuhan city. Then, in each district, 3 to 5 communities were randomly enrolled. For each community, 60 to 100 subjects were enrolled. Exclusion criteria were: age <65 years; no daily life activities; with dementia, sever bodily pain, congestive heart failure, dizziness, uncontrolled high blood pressure (exceeding 160/100 mmHg). Finally, a total of 2096 (1031 male and 1065 female) adults older than 65 years were included in our study.

The study was approved by the institutional review boards of Center for Disease Control and Prevention, Wuhan city and all subjects provided informed consent for participation. The methods were carried out in accordance with the principles of the Declaration of Helsinki.

The demographic characteristics (sex, age, height, weight, smoking status, yeas of education) were collected. In addition, Senior Fitness test and Mini-mental State Examination were used to measure FF and cognitive performance.

### Functional fitness measurement

FF was conducted by SFT which is developed for early identification of older people who are at risk of losing functionality. After 10 min of warm-up, participants were instructed by a trained instructor. SFT was then completed in the designed order: (1) 30s-chair stand: to assess lower body strength; (2) 30s-arm curl: to assess upper body strength; (3) Back scratch: to assess upper body (shoulder) flexibility; (4) Chair-sit-and-reach: to assess the flexibility of the lower extremities; (5) eight-foot-up-and-go: to assess agility/dynamic balance as an index of basic mobility skills and (6) 2 min- step: to assess aerobic endurance. Each test was strictly performed according to the SFT Manual^29^.

### Mini-mental state examination measurement

Cognitive assessment was evaluated by MMSE which has been proven as a reliable, sensitive and valid method for cognitive performance examining. We performed the Chinese version of MMSE by trained nurses. MMSE measured: (1) temporal and spatial orientation: 10 points; (2) immediate memory: 3 points; (3) recall: 3 points; (4) attention: 5 points and (5) language: 9 points. Total MMSE scores range from 0 to 30 points^30^. In Chinese population, it was suggested that CI should be defined as MMSE score <24 in people with education for less than 9 years, or <26 in those who have received formal education for more than 9 years^31^.

### Statistical analysis

Normality of distribution for continuous variables was tested by the Kolmogorov-Smirnov test. Normal distribution data were presented as mean ± SD, and the differences between males and females were compared by unpaired Student’s *t*-test. To explore the key sub-dimensions of FF and cognitive function influencing their overall relationships, we used canonical correlation analysis (CCA). This statistical method calculated the correlation between two sets of variables and generated statistically independent pairs of new variables, which were referred to as canonical variables. In this study, CCA was conducted using six FF variables as predictors of five MMSE sub-dimensions in males and females separately. *P* < 0.05 was accepted as statistically significant. Analyses were performed with SAS Software, Version 9.4 for Windows (SAS Institute Inc., Cary, NC, USA).

### Data availability statement

The datasets generated during and/or analyzed during the current study are available from the corresponding author on reasonable request.
